# Recurrent Bacteremia, a Complication of Cyanoacrylate Injection for Variceal Bleeding: Report of Two Cases and Review of the Literature

**DOI:** 10.1155/2009/407053

**Published:** 2009-08-16

**Authors:** T. Galperine, C. Flateau, M. D. Venon, F. X. Lescure, G. Béraud, T. Said Ibrahim, F. Delisle, F. Durand, K. Faure, G. Pialoux, B. Guery

**Affiliations:** ^1^Service des Maladies Infectieuses et Tropicales, AP-HP Hôpital Tenon, 75970 Paris cedex 20, France; ^2^Service des Maladies Infectieuses et Tropicales, Hôpital Huriez, CHRU Lille, 1 Place de Verdun, 59045 Lille Cedex, France; ^3^Laboratoire de Bactériologie, AP-HP Hôpital Tenon, 75970 Paris cedex 20, France; ^4^Service d'Hépatologie, AP-HP Hôpital Beaujon, 92110 Clichy cedex, France

## Abstract

We report the first description of recurrent bacteremia in two patients after cyanoacrylate injection for gastric varices bleeding treated with antibiotics alone. Adapted and prolonged antibiotic treatment allowed a complete resolution of the infection with no relapse after more than 6 months. According to recent data, prophylactic antibiotics should be further investigated for patients with bleeding varices undergoing cyanoacrylate injection.

## 1. Introduction

Local injection of N-butyl-2-cyanoacrylate, a propolymer which polymerizes immediately after blood contact, has been recommended for the treatment of gastric or oesophageal varices bleeding [1].

A bacteremia has been described in several reports following this procedure [2], but could also be related to the acute variceal haemorrhage itself [3]. This bacteremia is usually transient involving oral or digestive pathogens [2]. As opposed to these transient episodes, recurrent bacteremia is not frequently encountered [2, 4]. We here report two cases of recurrent bacteremia with septic complications after variceal cyanoacrylate injection.

## 2. Case 1

A 69-years-old man was admitted for fever, dyspnea, and abdominal pain. His past medical history only reported an idiopathic portal thrombosis with recurrent acute gastric bleeding. Six months before this admission, the bleeding was controlled with N-butyl-2-cyanoacrylate and ethiodized oil (6 mL, 1 : 1 ratio) (Laboratoire Guerbet, Aulney-sous-Bois, France) injection. The clinical examination recovered only a sensitive abdomen. C-Reactive Protein was measured at 330 mg/L, polymorphonuclear cells at 12 000/mm^3^. Chest CT showed a thrombus in the pulmonary arteries centered on a high-density image possibly corresponding to a foreign material. Abdominal CT scan showed a perigastric infiltration and cyanoacrylate material in the gastric wall. An empiric treatment with ceftriaxone was introduced and led to the improvement of the patient. *Micromonas micros* was isolated from two blood cultures, the treatment was switched to amoxicillin to reach a 2-week duration and the patient was discharged at home.

One month, later he was readmitted for recurrent postprandial sepsis with gastric pain and no other symptom. Laboratory parameters showed a white blood cell count of 29 200/mm^3^ with 19 000 neutrophils/mm^3^. *Micromonas micros* was once again isolated from blood cultures. Upper gastrointestinal endoscopy showed a 6 mm nonbleeding ulceration near gastric varices. Transthoracic and transoesophageal echocardiography were normal. Radio-labeled leukocytes scintigraphy showed a hyperfixation on the variceal plugs. Abdominal CT scan showed an abscess contiguous to gastric cyanoacrylate material. A surgical treatment was ruled out. As the fever persisted over one week of amoxicillin treatment (6 g per day), a switch to dalacin (2400 mg per day) with imipenem (4 g per day) was performed. Four days later, fever and abdominal pain resolved. After two weeks of intravenous antibiotic therapy, oral cefuroxil (1 g per day) was introduced. Abdominal CT scan after two months of treatment showed a complete regression of the gastric abscess. The antibiotic regimen was interrupted after 3 months, and one month later, radio-labeled leukocytes scintigraphy was normal. Six months later, the patient still presented no relapse.

## 3. Case 2

**Figure 1:**
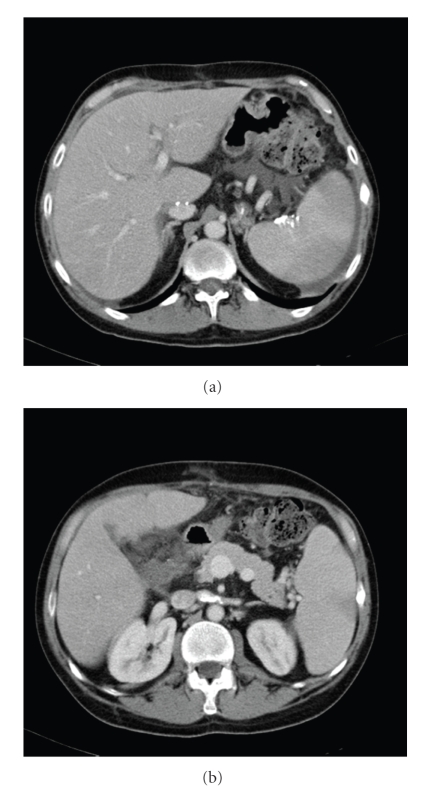
Thoracoabdominal computed tomography: (a) multiple splenic infarctions, (b) radio-opaque material in the left renal vein and the splenic hilum corresponding to cyanoacrylate emboli.

A 46-years-old human immunodeficiency virus- (HIV-) positive patient with a Child-Pugh grade C hepatitis B and delta virus cirrhosis was admitted for variceal bleeding with severe haemorrhage. He was successfully treated by variceal injection of N-butyl-2-cyanoacrylate and ethiodized oil (6 mL, 1 : 1 ratio) (Laboratoire Guerbet, Aulney-sous-Bois, France). Two weeks after the injection, he developed a fever with shivers and no other clinical signs which lead to hospital admission. Two blood cultures yielded a wild-type *Klebsiella pneumoniae*. No specific site of infection was found despite an evaluation including chest radiograph, transabdominal ultrasound examination, transthoracic echocardiography. Upper gastrointestinal endoscopy revealed no active bleeding. The patient was treated with piperacillin-tazobactam and ciprofloxacin for 15 days associated with gentamicin during the first 3 days. He underwent a liver transplantation one month later. Another episode of *K. pneumoniae* bacteremia occurred 10 days after the transplantation and was treated with the same antibiotic regimen for 15 days. Thoracoabdominal computed tomography (CT) revealed multiple splenic infarctions and radio-opaque material in the left renal vein and the splenic hilum corresponding to cyanoacrylate emboli (Figure 1). Following this episode, he remained under oral amoxicillin-clavulanate (3 g per day) to reach 6 weeks of antibiotic treatment. One month later, he was admitted for a third episode of *K. pneumonia* bacteremia. The analysis of the sensitivity of this pathogen revealed an intermediate susceptibility to amoxicillin-clavulanate and fluoroquinolones. An RFLP testing would have been interesting but was not performed at the time. Transthoracic and transoesophageal echocardiographies were normal. Thoracoabdominal CT and endoscopic examination were unchanged. An antibiotic regimen associating ceftriaxone 2 g and trimethoprim-sulfamethoxazole was introduced for three months. Ten months after the end of the treatment, there was still no recurrence.

## 4. Discussion

We report two cases of recurrent bacteremia after variceal cyanoacrylate injection combined with radio-opaque lipiodol for the treatment of gastric variceal bleeding. The frequency of this complication is unknown but remains probably low. Only few cases have been described in literature [2, 4]. Bacteremia is generally transient without clinical consequences. Several mechanisms of bacterial contamination have been reported [2, 5]. It could be related to a bacterial invasion through ruptured gastric variceal mucosa [5] with a secondary contamination of the injected material. Another possibility would be a bacterial contamination during cyanoacrylate injection [6], a potential transluminal seeding from the needle, a contamination of the side channel of the endoscope, or a contamination of cyanoacrylate itself. In all cases, consequences would be an infected N-butyl-2-cyanoacrylate conglomerate localized in the varices associated with an anatomic path between the gastrointestinal lumen and the vascular spaces [2]. In contrast to traditional sclerosing agents, cyanoacrylate has been recognized to have an in vitro antimicrobial effect against Gram-positive and Gram-negative bacteria [7, 8]. This finding was however never confirmed in vivo underlining the balance between a potential antimicrobial effect and the fact that cyanoacrylate is a foreign material and may, thereby, provide an ideal route for bacterial invasion.

As probably reported in the first case and in the literature, the communication between the gastrointestinal lumen and the vascular spaces leads to recurrent bacteremia [2, 4]. Another well-known complication is a glue embolization [9]. A concomitant infection, as reported in the second case, is exceptional [10], in our case, cyanoacrylate distal endovascular emboli lead to chronic *Klebsiella pneumoniae* bacteremia comparable to an intravascular foreign body infection. Consistent with literature, in our cases, the micro-organisms identified in the blood cultures are mainly oropharyngeal commensals and bacteria present in gastrointestinal tract [2]. Usually, removal of the foreign device is required although complete spontaneous expulsion of the cyanoacrylate conglomerates have also been described [2]. In both of our cases, surgery was not necessary and medical treatment with antibiotics alone was preferred based on extremely high operative risks. The duration of the antibiotic treatment is unknown but needs to be prolonged. These two cases are the first description of cyanoacrylate-related infection with a successful medical treatment and a follow up of more than six months. In a recent study, risk of significant bacteremia is related to the status of active or recent bleeding [5]. In these specific cases, especially in immunocompromised patients, this rare but severe infectious complication should lead to discussing the benefit of prophylactic antibiotics use although this is not routinely recommended.

## 5. Conclusion

Repeated bacteremia in a patient who has undergone cyanoacrylate injection should raise a strong suspicion of foreign-body-type infection of the polymerized N-butyl-2-cyanoacrylate. We report the first description with a favorable evolution after antibiotic treatment alone with a followup of more than 6 months. These two observations encourage further research to understand the epidemiology of these infections to help investigate the potential role of prophylactic antimicrobials.
